# Longitudinal effects of negative emotionality on loneliness in early childhood considering solitude preference and sibling status

**DOI:** 10.1038/s41598-025-86684-7

**Published:** 2025-03-27

**Authors:** Juanjuan Sun, Bowen Xiao, Yan Li

**Affiliations:** 1 College of Preschool Education, Shanghai Normal University, 100 Guilin Road, Xuhui District, Shanghai, China; 2 Psychology Department, Carleton University, Ottawa, Canada

**Keywords:** Negative emotionality, Solitude preference, Loneliness, Preschool children, Human behaviour, Psychology, Health care

## Abstract

As declining birth rates emerge as a significant societal concern, understanding the implications of being an only child versus having siblings is becoming increasingly important in China, especially in relation to social and emotional development. This longitudinal study examines the role of solitude preference and sibling status in the relationships between negative emotionality and loneliness in early childhood. The participants were 204 children (*M*_*age*_= 48.58 months, *SD* = 3.60). At Time 1, the children were interviewed to assess their preference for solitary play and loneliness, with a follow-up assessment of loneliness conducted at Time 2, two years later. Meanwhile, mothers provided evaluations of their children’s negative emotionality at Time 1. The findings indicate that negative emotionality positively predicts loneliness two years later, with solitude preference acting as a mediator in this relationship. Additionally, sibling status was found to moderate the link between negative emotionality and solitude preference. Specifically, for only children, higher negative emotionality intensified the relationship between solitude preference and loneliness, whereas having siblings acted as a protective factor, mitigating this effect. These results underscore the potential social and psychological disadvantages faced by only children and highlight the protective role siblings can play in mitigating feelings of loneliness.

## Introduction

The global decline in fertility rates has led to an increase in the number of only children, prompting growing interest in their unique developmental challenges^1^. Particularly, early childhood is a critical period for social and emotional development, yet only children may experience these differently compared to those with siblings^2^. Loneliness is a subjective experience of dissatisfaction with one’s social relationships, characterized by feelings of isolation, sadness, emptiness, and a desire for social connection^3^. Research has shown that loneliness emerges as early as preschool age^4^ and is closely associated with various developmental adaptation issues in children, such as depression, low self-esteem, and psychosomatic symptoms^5^.

Western studies have extensively explored the factors influencing children’s loneliness and its relationship with social adaptation^6^. However, in China, particularly during the preschool stage, the mechanisms underlying loneliness remain unclear. It is well-established that significant cultural differences exist between Eastern and Western societies^7^. For example, compared to individualistic societies, collectivistic societies place greater emphasis on interpersonal connections and the importance of “fitting in”^8^. Traditional Chinese culture places a strong emphasis on harmony and collective interests. In this cultural context, excessive self-focus and detachment from the group can negatively impact interpersonal relationships, as it goes against the values of unity and social cohesion^8,9^. In environments that place a strong emphasis on interpersonal interdependence, individuals may have a lower threshold for experiencing loneliness, as their sense of well-being and connection is closely tied to maintaining close social bonds and group cohesion^10^. Consequently, in collectivistic societies, children who display highly negative emotions in social interactions or spend more time alone may be more susceptible to experiencing loneliness. Given these cultural differences, it is crucial to investigate the unique manifestations and underlying mechanisms of loneliness in Chinese preschool children. Accordingly, the goal of the present study was to investigate how early negative emotionality affects loneliness through solitude preference in preschool children over a two-year longitudinal design, and how these relationships are moderated by the presence or absence of siblings.

### Negative emotionality and loneliness

The direct linear effects model indicates that temperament can directly influence children’s social adaptation, suggesting that children with extreme temperamental characteristics typically exhibit behavioral outcomes consistent with these traits^11^. Negative emotionality is a core component of early temperament, referring to a general tendency to experience negative emotions. Children with high levels of negative emotionality exhibit traits such as irritability, moodiness, difficulty in being soothed, and strong negative reactions^12^. Most studies have found that negative emotionality is significantly and positively correlated with internalizing problem behaviors such as anxiety and depression^13,14^. For example, research shows that higher scores on the fear dimension of temperament at age 2 can positively predict anxiety symptoms reported by mothers when the child is age 4^15^. This is attributed to the fact that children with high levels of fearful temperament are more likely to use avoidance strategies when regulating their emotions, leading to missed opportunities to make new friends in social interactions, thereby increasing the risk of subsequent anxiety. Children with high negative emotionality often show concern in interpersonal relationships, leading them to maintain psychological distance in social interactions as a way to avoid the risk of rejection. This tendency increases their vulnerability to loneliness and other negative emotional states^16^. Indeed, studies have found that children with high negative emotionality tend to exhibit lower social self-efficacy and greater feelings of loneliness^17^ which can even extend to difficulties in online interactions. Therefore, we expected that high negative emotionality would be positively associated with child’s sense of loneliness.

### The mediating role of solitude preference

Social relationships are universally acknowledged as a key component of a fulfilling life, playing a critical role in influencing both loneliness and subjective well-being^18^. According to the Social Needs Approach, loneliness arises when individuals perceive their social networks as inadequate in either quantity or quality^19^. Children who spend prolonged periods in solitude often struggle to effectively engage with social support networks, resulting in a significant lack of social support resources, making them more prone to feelings of loneliness. For instance, research by Coplan et al.^4^ indicates that children aged 3 to 7 who prefer solitary play are significantly and positively correlated with the amount of time spent alone and negatively correlated with perceived peer acceptance. As a result, children who prefer solitary activities may struggle to derive social satisfaction and positive emotions from peer interactions, leading to dissatisfaction with their social networks and a heightened sense of loneliness^6^. However, few studies have explored the relationship between young children’s preference for solitary play and loneliness, partly due to the lack of age-appropriate assessments of social preferences in early childhood. Our study utilizes the Preference for Solitary Play Interview (PSPI), a validated instrument that allows young children to reliably and accurately express their internal social motivations^20^, thereby deepening our understanding of their solitude preferences. This self-report approach overcomes the challenges of inferring children’s social motivations by providing direct insights into their preference for solitary play^6^. Using age-appropriate methodologies like the PSPI, our study offers more precise assessments of early childhood social preferences, which are essential for investigating their mediating role between negative emotionality and loneliness.

Furthermore, children’s temperament, particularly negative emotionality, is associated with lower social competence and behavioral problems^14^. The Emotional Reactivity Hypothesis suggests that children with aggressive, regressive, and reactive temperaments often have lower social-cognitive abilities^21^. Children with high negative emotionality are likely to have lower social self-efficacy, which leads them to avoid social activities and prefer being alone^22^. As a result of their preference for solitude, these children have fewer opportunities to engage with others, which hinders the development of their social skills and amplifies the negative effects of solitary preferences on their experience of loneliness^3^. As discussed before, in Western cultures, independence, autonomy, and confidence are valued, and solitude may be seen as a sign of autonomy and competence^23^. In contrast, Chinese culture, as a representative of Eastern cultures, places a stronger emphasis on interpersonal dependency and harmony. In this context, the excessive expression of negative emotions and prolonged solitude are often perceived as indicators of immaturity and inadequate social skills^7^. Consequently, we expected that children’s preference for solitude would mediate the relationship between negative emotionality and loneliness, with children exhibiting high negative emotionality being more likely to engage in solitary play, which, in turn, could lead to heightened feelings of loneliness.

### The moderation of sibling status

The relationship between negative emotionality and preference for solitude would be influenced by family structure, such as the presence of siblings. The stereotype that only children are at a “disadvantage” has long been prevalent^1^. With the implementation of China’s two-child policy in 2016, more families are having two children. For most individuals, siblings spend more time together than they do with their parents, making them a critical influence in personal development and socialization^24^. However, few studies have explored whether only children differ in the relationship between negative emotionality and preference for solitude. According to Social Learning Theory, siblings play a positive role in shaping child development. During childhood and adolescence, siblings are regarded as companions, friends, and role models^25^. Indeed, empirical studies have found that sibling relationships positively impact emotional understanding and interpersonal relationship quality, with children having siblings exhibiting higher emotional regulation abilities and levels of peer acceptance^26^. Through interactions with siblings, children have the opportunity to learn important skills such as cooperation and emotional understanding, which can help them develop more effective regulation strategies when dealing with negative emotions^24^. Thus, interactions with siblings may help mitigate the impact of negative emotionality on the preference for solitude. In contrast, only children often develop closer emotional ties with adults, which may lead them to choose solitude as a self-regulation strategy when facing negative emotions. The lack of opportunities for emotional regulation learning through sibling interaction may exacerbate the negative impact of negative emotionality on the preference for solitude in only children^6^.

While sibling status is hypothesized to moderate the relationship between negative emotionality and preference for solitude, its influence may be less pronounced in collectivistic societies like China. Strong ties with extended family members or peers often substitute for sibling relationships, offering opportunities for emotional learning and socialization^4^. These communal dynamics suggest that only children in collectivistic contexts may face fewer disadvantages, as broader social networks can buffer the impact of negative emotionality on solitude preference^1^. This study examines sibling status while considering these cultural nuances, providing insight into how temperament and family structure interact in collectivistic settings.

Therefore, we hypothesized that the presence of siblings would moderate the relationship between negative emotionality and the preference for solitude, with only children exhibiting a stronger preference for solitude due to a lack of sibling interactions that could buffer negative emotionality. This moderating effect is particularly significant in preschool children, who are in a critical developmental stage for acquiring social interaction skills and emotional regulation abilities. During this period, the presence or absence of siblings can profoundly shape a child’s socialization experiences and influence their socio-emotional development.

### The present study

Negative emotionality, a tendency to respond with negative emotions, can cause young children to feel marginalized in peer interactions, leading to a preference for solitude and loneliness. While research shows that loneliness can emerge in preschool years and remain stable, most studies focus on older children and rely on parent reports, with fewer investigations using self-reports from young children. Additionally, language ability plays a crucial role in determining the quality of self-reports, as children’s comprehension and communication skills directly affect their ability to articulate thoughts and experiences accurately^4^. To account for this, language ability was included as a control variable to ensure that the findings reflect the relationships between negative emotionality, preference for solitude, and loneliness without biases introduced by variations in language skills. This study addresses three key research questions: First, what is the relationship between negative emotionality and children’s feelings of loneliness? Second, do children with high negative emotionality exhibit a preference for solitude that worsens their feelings of loneliness, with solitude preference acting as a mediator? Finally, is the negative impact of temperament on solitude preference and loneliness more pronounced in only children? By exploring these questions, we aim to clarify the mechanisms through which negative emotionality contributes to loneliness in young children.

Based on existing research, we hypothesize: (1) parent-reported negative emotionality would positively predict child self-reported loneliness two years later; (2) Preference for solitude would mediate the relationship between negative emotionality and loneliness, specifically children with high negative emotionality are more likely to engage in solitary play, which can, in turn, lead to increased feelings of loneliness; (3) Sibling status would moderate the relationship between negative emotionality and preference for solitude. Specifically, compared to children with siblings, only children with high negative emotionality are expected to experience higher loneliness.

## Methods

### Participants

The 204 children participating in our longitudinal study were enrolled in 2 public preschools in Shanghai, China. Children’s ages ranged from 42 months to 54 months (M = 48.58 months, SD = 3.60 months). Among them, 112 were boys (54.90%), 152 were only child (74.51%). Primary caregivers’ education levels varied: 14.71% reported high school education or below, 70.09% held associate or bachelor’s degrees, and 15.20% had obtained master’s degrees or higher. Detailed demographic information is presented in Table 1.

**Table 1:** Basic family information (N = 204).

Variable	*n*	%
Child gender		
Boy	112	54.90
Girl	92	45.10
Sibling status		
Only children	152	74.51
Children with siblings	52	25.49
Mothers’ working status		
Full-time mother	41	20.10
Working mothers	163	79.90
Mothers’ educational degrees		
Senior school	30	14.71
Junior college	48	23.52
Bachelor’s degree	95	46.57
Graduate’s degree	31	15.20

### Procedure

Prior to data collection, the study received approval from the Research Ethics Committee (ethical approval number: 2020040). Researchers introduced the study objectives to parents through the participating kindergartens, obtaining informed consent from all parents of the participating children. All assessments were conducted individually in quiet rooms at the participating preschools by trained research assistants. As part of their involvement in the larger study, parents completed several questionnaires regarding themselves, their child, and their home environment. On the day of the assessment, the study details were explained again to the participants, with informed consent obtained from the parents and verbal assent from the children. Data were collected at two time points, approximately two years apart. At time 1 (T1), the children’s temperament, preference for solitary games, and sense of loneliness were evaluated through self-report from the mother and the child. At time 2 (T2), the children’s sense of loneliness and receptive vocabulary were re-evaluated.

### Measures

#### Negative emotionality

In this study, the child’s parent, typically the mother, completed the Very Short Form of the Children’s Behavior Questionnaire^27^. This instrument evaluates key temperament dimensions, including surgency, effortful control, and negative emotionality. Parents rated their children’s temperament on 7-point scale; 1 = extremely untrue of your child and 7 = extremely true of your child. Each dimension contains 12 items. An example item for negative emotionality “Seems to feel depressed when unable to accomplish some tasks.” The negative emotionality scale was used for the analyses, demonstrating a reliability coefficient of 0.710.

### Solitude preference

The Preference for Solitary Play Interview (PSPI) was administered by trained researchers to assess young children’s preference for solitary play. This tool was originally developed by Coplan et al. for use with preschool children^20^. The PSPI comprises 11 illustrated scenarios that depict various activities: constructive activities (e.g., LEGO), functional activities (e.g., sliding on a playground slide), dramatic play (e.g., dress-up games), and rule-based games (e.g., board games). During the interview, the experimenter presents each picture individually, describes the activity depicted, and asks the child whether they would prefer to engage in the activity alone (scored as 1) or with a peer (scored as 0). In this study, the internal consistency reliability of the PSPI was 0.841. A higher total score on the PSPI reflects a stronger preference for solitary play among young children.

### Loneliness

The Loneliness and Social Dissatisfaction Questionnaire (LSDQ) was administered by trained researchers to evaluate young feelings of loneliness and social dissatisfaction^28^ at T1 and T2. Children were requested to respond to 16 self-statements (e.g., Do you enjoy chatting with other children in kindergarten? ) using a 3-point scale ranging from 0 (Yes) to 2 (Never). To ensure comprehension among young children, the response options (“Yes,” “Sometimes,” and “Never”) were explained through examples in the instructions before the interview commenced. Once the children demonstrated an understanding of these options, the interview proceeded. In this study, the internal consistency reliability of the LSDQ was 0.823 at T1, 0.752 at T2. A higher total score indicates a higher level of loneliness experienced by the child.

### Language ability

The Peabody Picture Vocabulary Test (PPVT)^29^ is a measure of receptive vocabulary for children ages of 3–6. The experimenter stated a word, and children chose one of four pictures that best matched the word. The task continued until children answered incorrectly on 8 of 12 words in a set. The PPVT correlates highly with verbal intelligence measures such as the Wechsler Preschool and Primary Scales of Intelligence-Revised (WPPSI-R). We standardized each of these outcomes to have a mean of zero and standard deviation of one so that all of the results can be interpreted in standard deviation units. As this study relied on children’s self-reports, their language ability played a crucial role in determining the quality of the interviews, as it directly affected their understanding, expression, and ability to accurately convey their thoughts and experiences^20^. Therefore, language ability was included as a control variable.

## Results

### Preliminary analysis

Table 2 presents the means, standard deviations, and correlation coefficients of the main research variables. From Table 2, it can be observed that T1 negative emotionality is positively correlated with T1 and T2 loneliness, T1 solitude preference, and sibling status. Furthermore, T1 solitude preference is positively correlated with T1 and T2 loneliness.

**Table 2:** The descriptive statistics and intercorrelations for study variables.

	M	SD	1	2	3	4	5
1.T1 Negative emotionality	3.75	0.62	-				
2.T1 Solitude preference	1.80	2.42	0.186**	-			
3.T1 Loneliness	7.74	4.92	0.191**	0.315***	-		
4.T2 Loneliness	11.29	3.05	0.127*	0.174*	0.065	-	
5.Sibling status	1.26	0.42	0.139*	0.028	0.122	0.072	-

*p < .05, **p < .01, ***p < .001.

### Mediating model analysis

The mediation analysis was conducted following the procedure outlined by Hayes^30^, the relationship between the independent variable and the dependent variable was first examined. As shown in Table 3, after controlling for covariates, the results indicate a significant positive correlation between T1 negative emotionality and T2 loneliness (*β* = 0.171, *p* < .005). Thus, Hypothesis 1 was supported.

**Table 3:** The mediating effect of Solitude preference.

Predictors	Model 1 (T2 Loneliness)			Model 2 (T1 Solitude preference)			Model 3 (T2 Loneliness)		
	*β*	*t*	*95%CI*	*β*	*t*	*95%CI*	*β*	*t*	*95%CI*
Gender	0.08	1.15	(−0.058,0.217)	−0.15	−2.20*	(−0.268, −0.019)	0.10	1.48	(−0.034,0.241)
Age	−0.11	−1.58	(−0.243,0.027)	−0.08	−1.19	(−0.205,0.051)	−0.10	−1.40	(−0.229,0.039)
Education	0.11	1.34	(−0.050,0.260)	0.11	1.50	(−0.035, 0.258)	0.09	1.11	(−0.068,0.241)
Working status	−0.14	−1.73	(−0.289,0.019)	−0.03	−0.43	(−0.178,0.114)	−0.13	−1.68	(−0.282,0.022)
T1 NE	0.17	2.14*	(0.014,0.329)	0.16	2.17*	(0.015, 0.313)	0.15	181	(−0.013, 0.303)
T1 Loneliness	0.07	1.01	(−0.068,0.213)				0.03	0.36	(−0.119,0.172)
T2 LA	−0.16	−2.22*	(−0.296,−0.017)				−0.16	−2.23*	(−0.294, −0.018)
T1 SP							0.16	2.18*	(0.015, 0.310)
*R* ^2^	0.08			0.15			0.10		
*F*	2.33^*^			5.01^***^			2.67^***^		

NE = Negative emotionality; Language ability = LA; SP = Solitude preference.

Subsequently, the mediating variable was included in the model for further analysis. The PROCESS macro Model 4 was employed to test the mediating effect of solitude preference. The results reveal a positive relationship between T1 negative emotionality and T1 solitude preference (*β* = 0.164, *p* < .005), and a positive relationship between T1 solitude preference and T2 loneliness (*β* = 0.163, *p* < .005). Therefore, solitude preference mediates the relationship between negative emotionality and loneliness. Further mediation analysis demonstrates that the mediating effect of solitude preference is significant (mediator effect = 0.027, SE = 0.019, 95% CI = [0.003, 0.082]). Hence, Hypothesis 2 was confirmed.

### Moderated mediation analysis

**Fig. 1:**
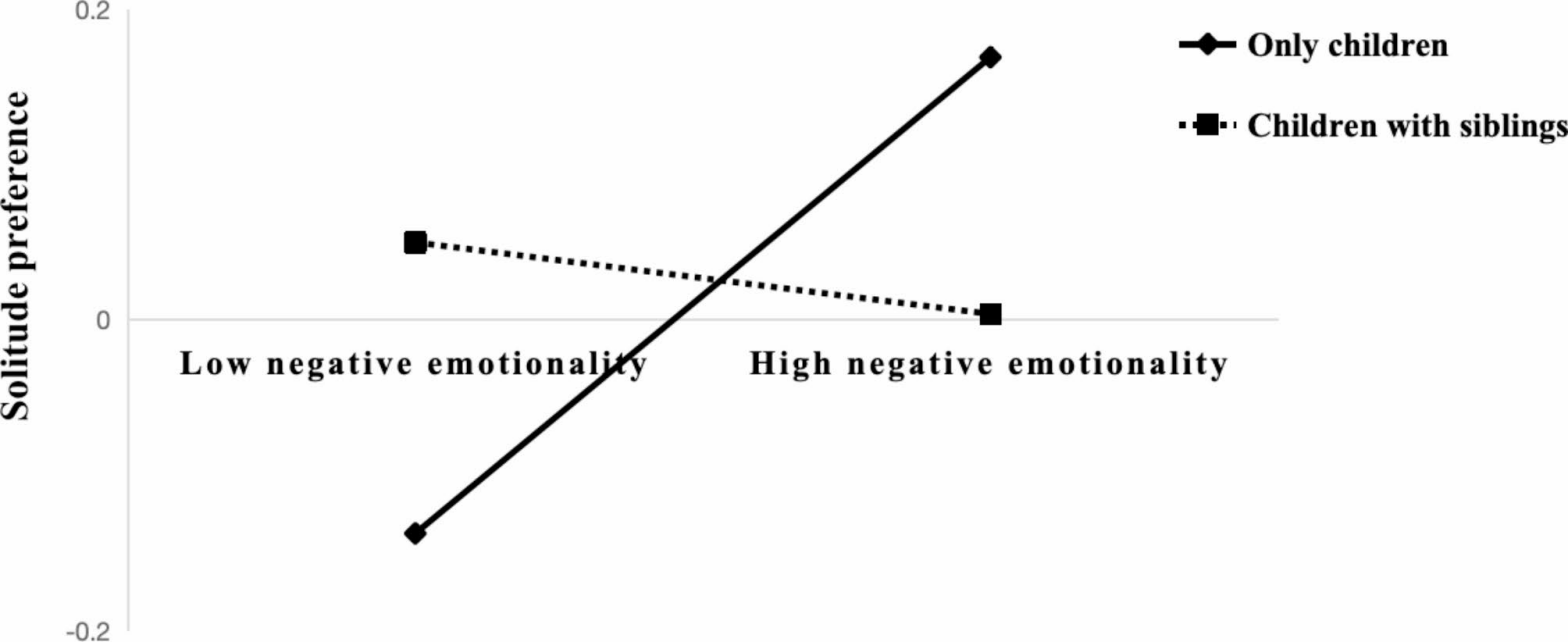
Moderating effect of sibling status on the relationship between negative emotionality and Solitude preference.

This study utilized the PROCESS macro Model 7 by Hayes^30^ to investigate whether sibling status moderates the relationship between negative emotionality and solitude preference. The results are presented in Table 4. The interaction term between negative emotionality and sibling status is significantly positively related to solitude preference (*β* = −0.176, *p* < .05). Thus, Hypothesis 3 was supported. The moderated mediation results are depicted in Fig. 1.

**Table 4:** Testing the moderated effect of sibling status.

Predictors	Solitude preference		
	β	t	95% CI
Gender	−0.15	−2.30*	(−0.279, −0.021)
Age	−0.09	−1.43	(−0.222, 0.035)
Education	0.10	1.34	(−0.047, 0.245)
Working status	−0.03	−0.40	(−0.176, 0.116)
T1 Negative emotionality	0.15	2.01*	(0.003, 0.304)
T1 Sibling status	0.01	0.15	(−0.129, 0.151)
T2 Language ability	0.01	0.09	(−0.119,0.131)
Negative emotionality* sibling status	−0.18	−2.35*	(−0.323, −0.028)
*R* ^*2*^	0.18		
*F*	4.59^***^		

To further explore how sibling status moderates the relationship between negative emotionality and solitude preference, simple slope analysis was used to illustrate the moderating effect of sibling status. As shown in Fig. 1, as children was only children, the detrimental impact of negative emotionality on solitude preference diminishes. When children were not only children, the influence of negative emotionality on solitude preference is no longer significant. This indicates that children with siblings can mitigate the adverse effects of negative affect on solitude preference.

In addition, sibling status can moderate the indirect pathway of negative effect on loneliness through solitude preference. As shown in Table 5, simple slope analysis revealed that when children are only children, the indirect effect of negative emotionality on loneliness through solitude preference is significant, indicating a negative impact of negative emotionality on loneliness via preference for solitude preference. However, when children had siblings, this indirect effect is no longer significant. Refer to Table 5 for details. Therefore, sibling status moderates the entire indirect pathway, showing that as the number of children increase, the negative impact of negative emotionality on loneliness through solitude preference diminishes.

**Table 5:** The mediating effect of solitude preference at different levels of sibling status.

	Sibling status	Effect	SE	95% CI
Solitude preference	Only children	0.043	0.026	(0.005, 0.108)
	Children with siblings	−0.003	0.017	(−0.042, 0.028)

## Discussion

In recent years, the unique social and emotional challenges faced by only children have garnered growing attention amid global trends of declining fertility rates. This study aims to longitudinally examine how negative emotionality in early childhood influences loneliness through solitude preference and to explore the moderating role of being an only child. Our results revealed that negative emotionality significantly predicts loneliness two years later, with solitude preference serving as a mediator in this relationship. Notably, the effect of negative emotionality on solitude preference was more pronounced for only children, suggesting that siblings may provide a protective influence, aiding in emotional regulation and mitigating the preference for solitude.

### The effect of negative emotionality on loneliness

First, we found that higher levels of negative emotionality in children can predict an increase in loneliness in preschoolers two years later, even after controlling for T1 loneliness.Consistent with previous research, children with high levels of negative emotionality are more likely to experience loneliness^22,31^. According to Gray^22^, the Behavioral Inhibition System (BIS) is closely linked to an individual’s negative emotionality. The BIS is sensitive to signals of punishment, non-reward, and novel stimuli, leading to the inhibition of behaviors associated with potential negative outcomes and the induction of negative emotions like fear and frustration. When this system is abnormally activated, individuals may experience heightened negative emotions, which can subsequently lead to problematic behaviors. Moreover, according to the diathesis-stress model, children with high negative emotionality have poor self-regulation abilities, making them more prone to experiencing negative emotions throughout life. When attempting to regulate these emotions, they often engage in repetitive rumination on negative feelings and outcomes, which can ultimately heighten their risk of developing depression and loneliness^32^. Indeed, research suggests that children with low levels of positive emotionality are less likely to use problem-solving strategies when dealing with negative emotions. Instead, they tend to rely on maladaptive strategies such as rumination, which can lead to increased feelings of loneliness^33^. Frequent experiences of negative emotions, such as anger and sadness, not only impede children’s cognitive processing but also limit their ability to regulate emotions effectively. This fosters the development of maladaptive emotional regulation strategies, which in turn can trigger internalizing problems like anxiety and loneliness. Evidently, elevated levels of negative emotionality impair emotional regulation and the strategies children employ, which over time become a significant risk factor for the development of loneliness.

### The mediation of solitude preference

Moreover, we found that children’s negative emotionality not only directly influences loneliness but also indirectly affects loneliness by increasing their preference for solitude. Specifically, children with high negative emotionality are more likely to engage in solitary play, which can, in turn, lead to increased feelings of loneliness. This may be because children with high negative emotionality often use passive regulation strategies^33^, such as avoidance and withdrawal, to manage negative emotions, resulting in extended periods of solitude. Consequently, they miss opportunities to make new friends during social interactions, increasing their subsequent risk of loneliness. For example, children with high levels of behavioral inhibition are more inclined to adopt passive emotional regulation strategies (such as prolonged staring at a broken toy rather than actively repairing it) and choose silence and avoidance in peer interactions. If children with high negative emotionality were to adopt more proactive regulation strategies, such as actively seeking help, they would likely show improved social competence two years later^34^. Prolonged solitude can hinder the development of social skills, such as reducing prosocial behaviors, and may result in children being perceived as socially withdrawn and rejected by their peers, ultimately increasing feelings of loneliness. For example, studies have shown that adolescents who frequently express negative emotions tend to score higher on depression scales^35^. Furthermore, heightened sensitivity and avoidance of novel stimuli can disrupt children’s normal emotional regulation processes, causing them to focus more on negative stimuli and, over time, contribute to loneliness.

### The moderation of sibling status

More importantly, this study found that sibling status moderates the relationship between negative emotionality and preference for solitude in young children. Specifically, the negative impact of negative emotionality on preference for solitude is more pronounced among only children, whereas the presence of siblings attenuates this negative effect. According to Social Learning Theory, only children may be more inclined to imitate adult behaviors, including ways of emotion regulation and coping with solitude. Without siblings, they may miss out on peer models for emotional expression and coping strategies, which could lead them to prefer solitude when dealing with negative emotions^24^. Indeed, research has indicated that only children might be at a disadvantage compared to those with siblings in decisively managing conflict, being described by peers as more passive, more victimized, and more aggressive^36^. In contrast, children with siblings have alternative models that provide diverse emotional coping strategies, such as alleviating emotions through partner play or shared activities. Since sibling relationships form a significant part of a child’s daily life, they offer numerous opportunities to practice these skills, which can then be applied to peer relationships^24^. From early childhood, children spend more time with siblings than with parents, and they engage in creative social role-play with siblings more frequently than with anyone else^37^. Research has shown that siblings can act as a buffer against stress^38^. As a result, children with siblings may have more opportunities for peer interactions, with siblings serving as sources of emotional support or providing distractions through interactive play. This dynamic can weaken the association between negative emotionality and a preference for solitude.

In addition, as discussed before, in collectivistic cultures like China, where group harmony and social cohesion are emphasized, alternative socialization mechanisms, such as close bonds with extended family or peers, can help mitigate the disadvantages of sibling absence^8^. However, the cultural focus on interdependence may also intensify challenges for children with high negative emotionality, as behaviors like prolonged solitude or poor emotional regulation may be viewed as socially inappropriate^9^. This dual influence of cultural norms and family structure can both buffer and amplify the social and emotional challenges faced by only children.

### Contribution and limitations

This study enhances our understanding of the relationship between negative emotionality and loneliness in children, particularly explaining why negative emotionality predicts a preference for solitude among only children but not among those with siblings. Firstly, it expands the existing literature on loneliness by focusing on preschool children, a demographic often overlooked in favor of school-aged children and adults. By employing a longitudinal design over two years, this research provides valuable insights into the temporal dynamics of negative emotionality and its impact on loneliness, highlighting how early emotional traits can predict later social outcomes. Secondly, the study utilizes self-reports from children to assess their preference for solitude and feelings of loneliness, offering a novel methodological approach that contrasts with the predominant reliance on adult reports in child research. This approach provides a more direct understanding of children’s subjective experiences. Lastly, the study sheds light on the developmental context of only children, revealing that the absence of siblings may exacerbate the pathway from negative emotionality to loneliness via an increased preference for solitude. This finding underscores the unique social challenges faced by only children in low fertility contexts.

Despite its contributions, this study has several limitations. First, the study was limited in its scope by considering only whether a child had siblings, without delving into the nuances of sibling relationships or birth order, both of which might significantly impact children’s peer interactions^39^. Future research should incorporate these nuanced sibling factors to more thoroughly understand the complex interplay between negative emotionality, solitude preference, and loneliness. Additionally, the study’s focus on a specific cultural and geographical context may limit the generalizability of the findings to other populations. Further research should explore these dynamics in diverse cultural settings to assess the universality of the observed effects. Furthermore, the findings also highlight the need for future research to explore how cultural norms and practices shape the developmental trajectories of children’s emotional regulation and social behaviors. For example, examining the role of extended family or community support networks in collectivistic societies could provide deeper insights into how non-sibling interactions influence the relationship between negative emotionality, solitude preference, and loneliness. Similarly, comparative cross-cultural studies could investigate how these dynamics differ between collectivistic and individualistic societies, offering a more nuanced understanding of the interplay between cultural values, family structure, and child development. Lastly, while the longitudinal design is a strength, the direction of this influence could be bidirectional, as emphasized in Bandura’s social cognitive theory of reciprocal determinism^40^. Future research could further examine the systematic changes in individuals with negative emotionality and their preference for solitary play over time, as well as the causal relationships between negative emotionality, solitary play preferences, and loneliness, thus elucidating the mechanisms of these relationships. This would provide empirical evidence to support intervention research aimed at alleviating early childhood loneliness.

### Practical implications

The findings of this study have significant implications for early childhood education and parenting practices. Educators and parents should be aware of the potential for negative emotionality to lead to increased loneliness over time, particularly in only children. Interventions aimed at fostering social skills and providing opportunities for peer interaction could be beneficial in mitigating these effects. Additionally, understanding the protective role siblings can play suggests that creating environments where only children can engage in sibling-like interactions might help buffer against the development of loneliness. Policymakers and educators should consider programs that promote social integration and emotional regulation skills from an early age to support the well-being of all children, especially those without siblings.

## Conclusion

This longitudinal study elucidates the intricate relationships between negative emotionality, preference for solitude, and loneliness in preschool children, with a particular focus on the moderating influence of sibling status. Our findings demonstrate that early negative emotionality significantly predicts loneliness two years later, with solitude preference serving as a crucial mediator in this relationship. Importantly, this mediation is moderated by whether a child is an only child. Unlike children with siblings, only children are more susceptible to the pathway from negative emotionality to increased solitude preference and subsequent loneliness, highlighting a potential vulnerability in their emotional and social development. The study advances the literature by focusing on loneliness in a younger demographic, utilizing child-reported measures rather than adult reports, and emphasizing the unique challenges faced by only children in low fertility contexts. These findings are significant as they suggest that siblings may play a protective role against the social withdrawal driven by negative emotions.

## Data Availability

The datasets used and/or analyzed during the current study are available from the corresponding author upon reasonable request.
